# A high-performance polymer composite column for coronavirus nucleic acid purification

**DOI:** 10.1038/s41598-024-51671-x

**Published:** 2024-01-11

**Authors:** Akli Zarouri, Aaron M. T. Barnes, Hamada Aboubakr, Vinni Thekkudan Novi, Qiuchen Dong, Andrew Nelson, Sagar Goyal, Abdennour Abbas

**Affiliations:** 1 Department of Bioproducts and Biosystems Engineering, University of Minnesota Twin Cities, 2004 Folwell Ave, Saint Paul, MN USA; 2 Division of Molecular Pathology and Genomics, Department of Laboratory Medicine and Pathology, University of Minnesota Twin Cities, 420 Delaware Street SE, Minneapolis, MN USA; 3 Department of Microbiology and Immunology, University of Minnesota Medical School, 689 23rd Ave SE, Minneapolis, MN USA; 4 Department of Veterinary Population Medicine, University of Minnesota Twin Cities, 1333 Gortner Ave., Saint Paul, MN USA

**Keywords:** Biological techniques, Molecular biology, Microbiology, Infectious-disease diagnostics, Virology, Analytical chemistry

## Abstract

Here, we report the development of a novel polymer composite (PC) purification column and kit. The performance of the PC columns was compared to conventional silica gel (SG) columns for the purification of nucleic acids from coronaviruses, including SARS-CoV-2, in 82 clinical samples. The results shows that PC-based purification outperforms silica gel (SG)-based purification by enabling a higher sensitivity (94%), accuracy (97%), and by eliminating false positives (100% specificity). The high specificity is critical for efficient patient triage and resource management during pandemics. Furthermore, PC-based purification exhibits three times higher analytical precision than a commonly used SG-based nucleic acid purification thereby enabling a more accurate quantification of viral loads and higher reproducibility.

## Introduction

The emergence of coronavirus disease 2019 (COVID-19) pandemic represents an unprecedented global challenge, with far-reaching consequences for societies around the world. The spread of the COVID-19 viral etiology, human severe acute respiratory syndrome coronavirus 2 (SARS-CoV-2), by respiratory droplets and passive contact poses an increased risk in densely populated environments, such as transportation hubs hosting millions of people^1,2^. Governments around the world have put in place a series of preventive measures and tools to curb viral transmission and prevent the appearance of successive waves^3^. One of the critical tools in the fight against the COVID-19 pandemic is the rapid and accurate detection of the causative agent, SARS-CoV-2, which enables early prevention of outbreaks in communities and hospitals. Currently, real-time quantitative Polymerase Chain Reaction (qPCR) is the gold standard in diagnostics and detection of viral disease etiology, including SARS-CoV-2^4,5^, as recommended by the World Health Organization (WHO)^4–6^. This is due to the high sensitivity and specificity of the qPCR compared to other viral detection methods such as viral antigen detection, standard plaque assay, serology, or CRISPR-based techniques^7–10^. However, qPCR performance is inherently tied to the quality and quantity of the nucleic acid extract present in the sample. Even slight variations in quality can lead to misleading results, including both false negatives and false positives^11^. The accuracy of the test is intricately linked to the efficiency of the viral genome extraction and purification processes^12^. The nucleic acid purification techniques that are currently used for this purpose suffer from low specificity (more false positives) and/or sensitivity (more false negatives). The implications of these elevated false negative rates may create considerable obstacles in effectively curbing the spread of viral infections^13^. Silica gel spin columns and magnetic beads have been commonly employed for nucleic acid extraction. Silica gel is negatively charged and the adsorption of the negatively charged nucleic acid macromolecule to the silica gel surface requires a positively charged binding agent which forms a complex with both nucleic acid and silica gel^14^. This is facilitated by a high concentration of chaotropic salt, to which the biological samples are exposed during the nucleic acid extraction process^15,16^. The salt acts as a bridge between the nucleic acid backbone and the silica surface by forming a layer of positive ions^17^.

Nevertheless, silica-based purification systems face limitations due to the strong binding affinity of smaller nucleic acid fragments. This compromises the overall binding efficiency to the silica matrix and renders it non-reusable. Furthermore, the extraction of these smaller fragments becomes increasingly challenging due to this strong binding interaction^18,19^. Silica gel columns also suffer from low nucleic acid recovery rate for samples containing lower than 1 μg of total nucleic acid. In such cases, the silica gel membrane needs a carrier nucleic acid to improve the yield^20^, which increases costs. Extraction using magnetic beads on the other hand faces challenges with interferences in PCR amplification and can be labor intensive^18^. While labor can be reduced using automated systems, it is still difficult to use magnetic separation for samples with large volumes (> 10 mL) due to limitations in the space distribution of the electromagnetic field^21^. Such limitations have made the testing process time consuming and costly, especially in the case of wastewater-based epidemiology.

The use of filter paper has been reported as a viable alternative to silica-based materials for nucleic acid purification from diverse sources^22^. However, it should be noted that within the paper, the accessibility of hydroxyl groups (OH) on the surface of cellulosic chains is limited, as some are inward-facing and not easily accessible^23^. Furthermore, the availability of surface OH groups on cellulose fibers typically ranges from 1 to 3% of the total hydroxy groups present in the original cellulose sample^24^. Consequently, the functionalization of fibers with TEOS provides a promising approach to enhance the availability of OH groups. This, in turn, enables a more efficient uptake of nucleic acids through the positive salt bridge formed between the OH groups and nucleic acid molecules.

This study introduces an innovative polymer composite filter tailored for RNA extraction and purification. The filter is based on a microporous cellulose paper functionalized with tetraethyl orthosilicate (TEOS) and optimized for the binding and easy elution of nucleic acids. The primary objective of this study is to evaluate the effectiveness of the polymer composite column in purifying coronavirus nucleic acids. This involves a comparative study between commercially available silica gel (SG) columns, including their extraction kits, and the polymer composite (PC) columns, coupled with a lab-made reagent kit.

Initially, the comparison was conducted using transmissible gastroenteritis virus (TGEV) as a lower biosafety level animal coronavirus surrogate for SARS-CoV-2. Subsequently, a full-scale comparative study was independently conducted at the University of Minnesota Medical School between SG columns and PC columns, each paired with their respective reagents. This study utilized clinical samples for the detection of SARS-CoV-2.

## Results and discussion

### RT-qPCR detection and quantification of TGEV

A preliminary feasibility study was conducted to confirm the functionality of both the SG and PC purification methods and associated kits for detecting the TGEV, preceding a full-scale study to evaluate analytical parameters such as sensitivity, specificity, and limit of detection. The results from the feasibility study show that both the SG and PC-based kits offer reliable detection and quantification of TGEV, as shown in Supplementary Fig. a. Building upon these positive results, subsequent in-depth studies were carried out to meticulously evaluate and compare the performance of the two nucleic acid extraction and purification methods.

### RT-qPCR detection and quantification of SARS-CoV-2

The COVID-19 Diagnostic Laboratory at the University of Minnesota conducted both preliminary and full-scale investigations aimed at the extraction and detection of SARS-CoV-2 RNA from samples obtained from COVID-19 patients. The quantification of SARS-CoV-2 samples collected from infected patients employed RT-qPCR and utilized RNA extracted through both the PC-based kit and the SG-based kit. The efficiency of these kits in extracting viral RNA was assessed across five analytical parameters: sensitivity, specificity, limit of detection (LOD), accuracy, and reproducibility. The preliminary study involved 16 clinical samples, and to adhere to FDA assay validation requirements, the full-scale study incorporated 32 positive samples collected from COVID-19 patients and 32 negative control samples (Refer to Table 1).

**Table 1:** Comparison of the clinical specificity and sensitivity of nucleic acid extraction kits using silica gel (SG) or polymer composite (PC)–based purification.

	Preliminary study (16 samples)		Full scale study (66 samples)	
	SG-based purification	PC-based purification	SG-based purification	PC-based purification
No. of true positives	12	13	30	30
No. of false positives	0	0	5	0
No. of true negatives	2	2	27	32
No. of false negatives	2	1	2	2
Sensitivity (%)	85.71	92.86	93.75	93.75
Specificity (%)	100	100	84.38	100
Accuracy (%)	88	94	89	97

### Clinical diagnostic sensitivity, specificity, and accuracy

1 $$Assay \,\,sensitivty=\frac{TP}{TP+FN}$$

2 $$Assay \,\,specificity=\frac{TN}{TN+FP}$$

3 $$Assay \,\,accurcy=\frac{TN+TP}{TN+FP+FN+FP}$$

Where TP refers to true positives, denoting correctly identified instances, while FN represents False Negatives, signifying instances erroneously classified as negative despite being positive. Additionally, TN stands for True Negatives, indicating correctly identified negative instances, and FP refers to False Positives, representing instances wrongly classified as positive despite being negative.

**Figure 1:**
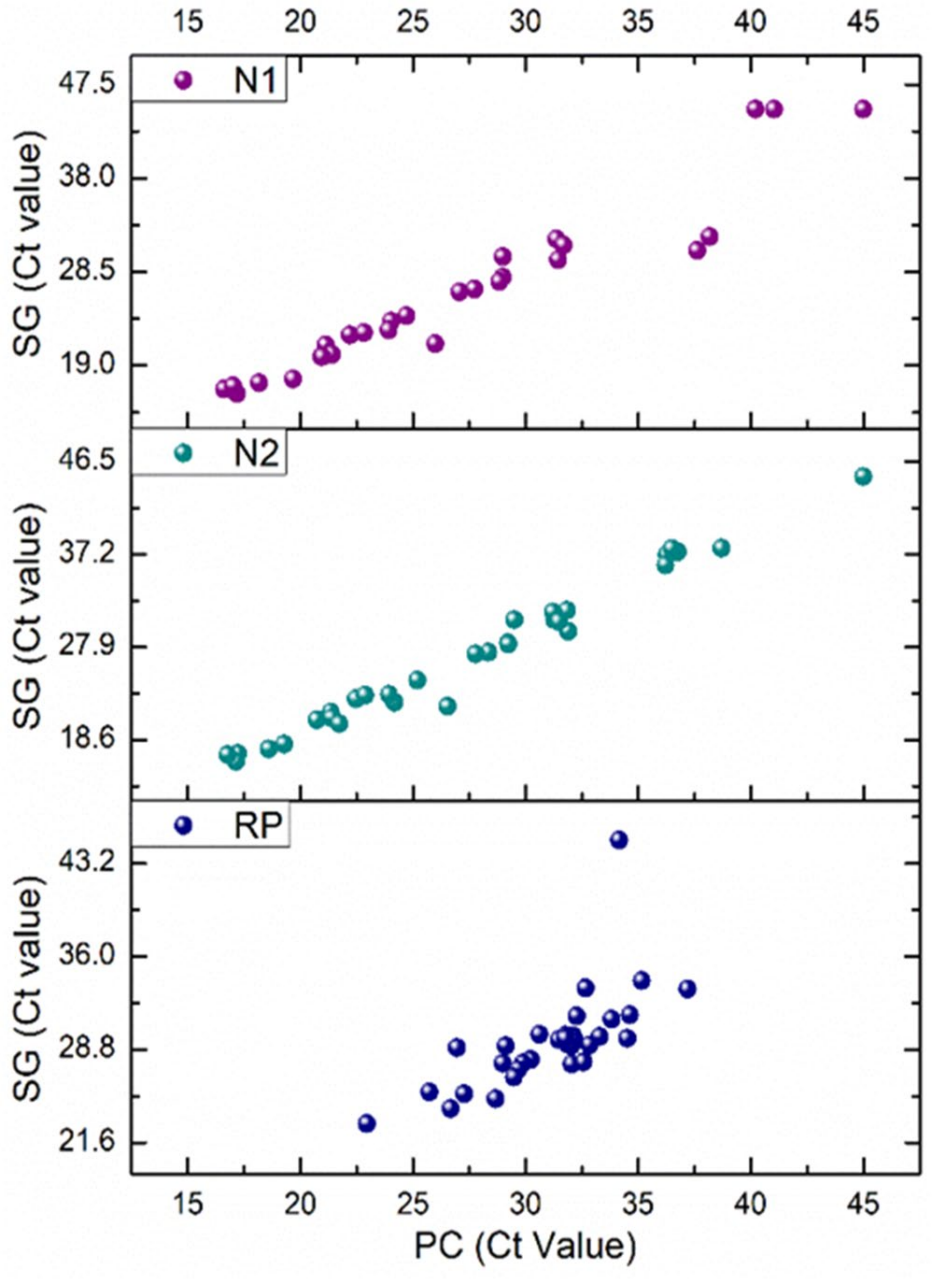
Comparison of Ct values obtained using SG- vs. PC-based purification kits for RT-qPCR detection of SARS-CoV-2.

Figure 1 shows a good linearity indicating an agreement between the Ct values obtained with SG-based kits and those obtained with PC-based kits.

The sensitivity, specificity, and accuracy values, calculated using Eqs. (1), (2) and (3), are detailed in Table 1. The results reveal that the sensitivity of detection improved by using the PC-based kit as compared to the widely used SG-based kit. Likewise, the specificity of RNA detection drastically improved up to 100% when the PC-based kit was used as compared to the SG-based kit, which only showed around 84% specificity. This means that PC-based purification can eliminate false positives, which is crucial for patient triage during pandemics. In addition to the improved accuracy by 8%, the PC-based purification demonstrated minimal variation, with only a 0.89% change in sensitivity and no variation in specificity observed between the preliminary study using the TGEV and the full-scale studies for SARS-CoV-2, indicative of consistent and reliable performance. In contrast, SG-based purification showed variations of 8% and 15.6% in the two trials, respectively. This difference suggests that the PC-based kit exhibits lower susceptibility to variations arising from diverse sample sources, users, and experimental conditions. This is partly due to the higher analytical precision (i.e. the ability to differentiate smaller changes in viral loads) as discussed below. The raw data for sensitivity and specificity calculations are available in the supplementary information, Tables a and b.

### Limit of detection (LOD)

**Figure 2:**
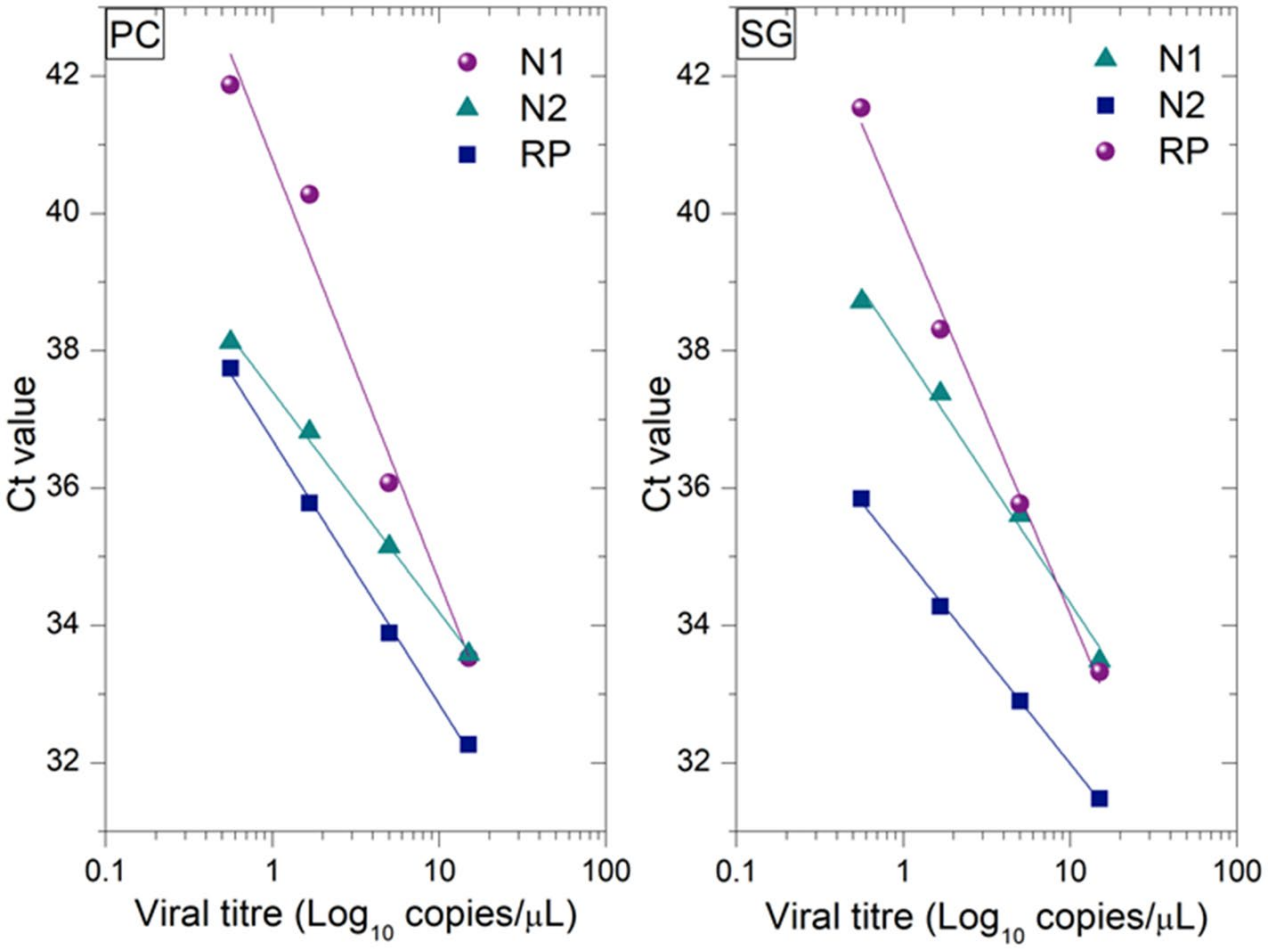
Comparison of the detection limits and Ct values of the RNA extraction kits, PC and SG in detecting SARS-CoV-2. The X axis is in the logarithmic scale. This figure is also used to calculate the analytical precision from the slope of the linear fit.

The limit of detection (LOD) was calculated based on the RT-qPCR results obtained from both the PC-based kit and the SG-based kit. The results, depicted in Fig. 2, demonstrate comparable limits of detection (LOD) of approximately 0.57 copy/µL (2.85 copies/reaction) for both kits, falling within the typical 5% standard deviation observed in most commercial extraction kits. Notably, our PC-based kit displays a significantly lower LOD compared to other viral RNA extraction methods proposed during the pandemic, which ranged from 10 to 50 copies per reaction^25,26^. Supplementary Information, Table c, contains the raw data used for LOD analyses. While the LOD of our PC kit was comparable to the commercial SG kit, Fig. 2 illustrates a significant difference in analytical precision, as evidenced by the slopes of the linear fits. This discrepancy underscores the kits’ capacity to discern smaller variations in viral loads. Upon examining Table 2, it is apparent that a 1 copy/µL alteration in RNA concentration induces a 0.28 change in Ct values for PC-based purification, compared to a mere 0.09 change for SG-based purification. This indicates that the PC-based kit exhibits at least three times higher analytical precision than the commonly used SG-based kit.

**Table 2:** Limits of detection, reproducibility and analytical precision of SG and PC kits.

	SG	PC
LOD (copies/ µL)	0.57	0.57
Analytical precision	0.09 Ct value per 1 RNA copy/µL	0.28 Ct value per 1 RNA copy/µL

## Materials and methods

### Materials and reagents

Whatman filter paper grade 5 was purchased from Cytiva (Marlborough, MA, USA). Tetraethyl orthosilicate (TEOS), iminodiacetic acid (IDA) 98%, guanidine thiocyanate (GuTC) and guanidine hydrochloride (GuHCl) were obtained from Sigma-Aldrich (Saint Louis, MO, USA). Empty spin columns with O-rings and collection tubes were purchased from Shanghai Perfect Foreign trade (Shanghai, China). dsDNA assay kit was purchased from Invitrogen (Carlsbad, CA, USA). The RNA assay kit was purchased from Invitrogen (Carlsbad, CA, USA). Reagents for TGEV propagation including Eagle's Minimum Essential Medium, Corning® MEM and Dulbecco’s Modified Eagle Medium Corning® DMEM were purchased from Mediatech Inc. (Manassas, VA, USA), 8% fetal bovine serum (FBS) and 1 × antibiotic–antimycotic were purchased from Gibco by Life Technologies (Carlsbad, CA, USA), bovine serum albumin solution- fraction V (7.5% BSA) was purchased from Thermo Fisher Scientific (MA, USA) and 0.65 U/mL TPCK-trypsin was purchased from Worthington biochemical Inc. (NJ, USA). PCR primers and probe were manufactured by Integrated DNA Technologies (IDT Inc., IA, USA). AgPath-ID One-Step RT-qPCR kit and ABI MicroAmp® Fast Optical 96-Well Reaction Plates were purchased from Applied Biosystems by ThermoFisher Scientific (CA, USA). Matrix gene transcript RNA was obtained from the University of Minnesota Veterinary Diagnostic lab.

The nucleic acid extraction kits QIAamp® DSP Viral RNA Mini Kit (number 52906; SG-based) was purchased from Qiagen (Germantown, MD, USA).

### Filter functionalization

The functionalization of Whatman filter paper was accomplished using TEOS (Tetraethyl Orthosilicate). Throughout various studies, TEOS has been employed either independently or in conjunction with other chemicals to strengthen paper or to pre-functionalize cellulose nanocrystals^27,28^. In this study, TEOS is employed to enhance the accessibility of hydroxyl groups (OH), playing a pivotal role in facilitating the purification of nucleic acids.

A precise solution of TEOS was prepared by combining TEOS, ethanol, and water in a well-defined molar ratio of (0.5:25:8). To conduct the functionalization, the filter paper was introduced into a porcelain Buchner funnel equipped with a perforated plate. The solution was then suctioned through the filter, ensuring optimal polymer density and filter porosity. During this process, the hydrolysed TEOS underwent silanol condensation, establishing covalent bonds with the cellulose hydroxyl groups and resulting in a thin, uniform coating on the filter paper. This interaction between TEOS and cellulose generated a chemical bond and stable reactive layer.

### Propagation of viruses

Transmissible gastroenteritis virus (TGEV) was propagated and titrated in *Sus scrofa* testis (ST) cells. The cells were grown in Eagle's Minimum Essential Medium supplemented with 8% fetal bovine serum (FBS), and 1 × antibiotic–antimycotic. For virus propagation, the ST monolayer (80–90% confluency) was infected with the TGEV at 0.1 multiplicity of infection (m.o.i.) and maintained in Dulbecco’s Modified Eagle Medium supplemented with 2% FBS and 1 × antibiotic–antimycotic. The infected cultures of TGEV were incubated in 5% CO_2_ incubators at 37 °C for 3–5 days until cytopathic effects (CPE) were observed under an inverted microscope. The virus was harvested by only one cycle of freeze and thaw at − 80 °C, followed by centrifugation at 3000×*g* for 10 min to pellet and discard the cell debris for partial purification. The propagated virus stocks of TGEV were aliquoted and stored at − 80 °C until used in the experiment.

### Virus titration

The 50% tissue culture infective dose (TCID_50_) method was used to titrate the virus in its host cell monolayers. Serial tenfold dilutions of the virus were prepared in the maintenance medium for the host cell described above. Confluent monolayers of the host cell prepared in 96-well plates were infected with 100µL of the virus dilution using 3 wells per dilution. The infected plates were incubated at 37 °C in a 5% CO_2_ incubator. The cytopathic effects (CPE) of the infectious virus were observed under an inverted microscope after 5 days. The titer of the viruses was determined by a previously developed method and expressed as TCID_50_/mL^29^.

#### RT-qPCR for TGEV quantification

PCR primers and probe set shown in Table d in the supplementary information were used^30^. The RT-qPCR primers were designed to target a conserved 146 bp region (corresponding to the region between nucleotides 370 and 515 of the TGEV S gene open reading frame, with reference to the sequence of TGEV-GenBank accession no. KX900410.1). The reactions were performed using the AgPath-ID One-Step RT-qPCR kit. The reaction mixture (25 μL) consisted of 5 μL of template RNA, 12.5 μL of 2 × RT-qPCR buffer, 1 μL 25 × RT-qPCR Enzyme Mix, 0.5 μL of 10 μM solutions of both TGEV-forward and reverse primers (200 nM final concentration), 0.30 μL of 10 μM probe solution (120 nM final concentration), and 5.20 μL of nuclease-free water. The RT-qPCR was performed in a QuantStudio‐5 Real‐Time PCR thermocycler system (Thermo Fisher Scientific-Applied BioSystems). The thermal cycling conditions were 45 °C/10 min for reverse transcription (RT), 95 °C/15 min for Taq polymerase activation, and 45 PCR amplification cycles using a 94 °C/1 s denaturation step and an annealing step of 58 °C/45 s. In each run of RT-qPCR, standard curve samples and no template control were used as positive and negative controls, respectively.

#### Calibration curves of TGEV RT-qPCR

The TGEV PCR standard/calibration curve was constructed for absolute quantification of viral genome copy number. Ten-fold serial dilutions of a 557 bp RT-qPCR purified amplicon of TGEV S gene (including the 146 bp target sequence of the RT-qPCR primer/probe set) were used. The 557 bp TGEV S gene fragment was produced by RT-qPCR reaction using an in-house developed primer set shown in Table d in the supplementary information. A 557 bp PCR amplicon with known copy number was used. Results were expressed as cycle threshold (Ct) values. The Ct values were used along with the standard curve to calculate the absolute genome copy number of TGEV, expressed as genome copies per mL.

### RT-qPCR for SARS-CoV-2 quantification

RT-qPCR using the standard US CDC primer–probe set for SARS-CoV-2 (N1 and N2 viral targets; human RNase P (RP) control) and operating under an FDA Emergency Use Authorization was done via the CLIA lab at the University of Minnesota Genomic Center^17^. Samples were defined as positive for SARS-CoV-2 if either N1 or N2 exceeded the clinical thresholds (CT < 40 cycles). All samples required detection of the RP control (CT < 40) to meet criteria. Samples extracted using both the PC-based kit and the control SG-based kit were quantified through this method.

### Viral sample collection

Clinical nasopharyngeal swab specimens from routine COVID-19 testing were collected by a health care provider and transported in viral transport medium (VTM) or universal transport medium (UTM). Fresh, refrigerated residual material from these collections was used for all extraction and molecular testing. All samples were obtained from the University of Minnesota Medical Center – Fairview system under Common Rule exemption.

### RNA extraction

Sample processing was conducted with both the SG and PC-based kits. A graphical protocol diagram for the PC-based nucleic acid (NA) extraction kit is shown in Fig. b in supplementary information. Briefly, a 100 µl aliquot of VTM/UTM was added to 560 µl of buffer LB containing carrier RNA in a microcentrifuge tube and incubated at 56 °C for 20 min to inactivate any virus present in the samples. After centrifugation (18,000×*g*; 1 min) the supernatant was centrifugally loaded onto a minispin column (850×*g*; 1 min), washed with buffer (WB; 500 µL) and dried via extended centrifugation (18,000×*g*; 3 min). The bound RNA was eluted (EB, 1 min incubation; 4500×*g*; 1 min) and the extracted samples were transferred for RT-qPCR. Some of the samples were also extracted using the SG-based kit as control to compare the efficiency of the PC columns and buffers in extracting nucleic acids.

### Limit of detection (LOD)

To allow for quantitative determination of the limit of detection (LOD) of the assay using RNA extractions from each column type, a synthetic SARS-CoV-2 standard control manufactured by Exact Diagnostics (EDx; #COV019) at 200 cp/µL viral nucleic acid and 75 cp/µL human gDNA was diluted into the provided EDx negative control (human gDNA only; EDx: #COV000) and serial dilutions were prepared: 90 copies/µL (Cp/ µL), 45 Cp/ µL, 15 Cp/ µL, 5 Cp/ µL, 1.67 Cp/µL, and 0.56 Cp/µL. (These EDx controls are manufactured to serve as a synthetic spike-in source for assay validations: copy number is standardized via ddPCR by the manufacturer)^17^. LOD experiments were run in triplicate for most dilutions (Supplementary information Table c).

### Biosafety & institutional control

Extractions and processing of infectious viral samples were carried out under BSL-2 + conditions (standard BSL-2 conditions with the addition of some BSL-3 practices such as using extra personal protective equipment). All experimental protocols, including safety and regulatory protocols are approved by the University of Minnesota Institutional Biosafety Commission. All methods were carried out in accordance with relevant guidelines and regulations. Informed consent was obtained from all subjects and/or their legal guardian(s).

## Conclusions

This study introduces an innovative kit designed for the extraction and purification of coronavirus RNA, featuring a novel polymer composite column paired with lab-made reagents. In a head-to-head comparison with commercially available kits relying on conventional silica gel columns, our novel polymer composite-based kit demonstrated significantly superior performance in detecting SARS-CoV-2 RNA in clinical samples. Notably, the PC-based kit exhibited comparable sensitivity to its SG-based counterpart while achieving a substantial 15.6% increase in specificity. This enhancement is pivotal in minimizing false positives during patient diagnosis. Additionally, despite both methods sharing a similar detection limit (0.57 copies/µL), the RT-qPCR assay’s analytical precision proved to be three times higher when utilizing samples extracted and purified with the PC-based kit. This notable advancement contributes to heightened accuracy and result consistency across diverse experimental conditions.

### Supplementary Information

Supplementary Information.

## Data Availability

Raw data and datasets generated and/or analysed during the current study are available in the supplementary information. Further data or additional information can be obtained upon request by contacting the corresponding author via email at: aabbas@umn.edu.
