# Supplementary material for: A high-performance polymer composite column for coronavirus nucleic acid purification
Source: Sci Rep. 2024 Jan 11;14:1138. doi: 10.1038/s41598-024-51671-x (PMC10784286; doi:10.1038/s41598-024-51671-x)
Supplement: Supplementary file 1 — Supplementary Information. [file 41598_2024_51671_MOESM1_ESM.docx]

**Supplementary Information**

A High-Performance Polymer Composite Column for Coronavirus Nucleic Acid Purification

Akli Zarouri,^1^ Aaron M. T. Barnes,^2,3^ Hamada Aboubakr,^1,4^ Vinni Thekkudan Novi,^1^ Qiuchen Dong,^1^ Andrew Nelson,^2^ Sagar Goyal,^4^ and Abdennour Abbas^1,^*

^1^Department of Bioproducts and Biosystems Engineering, University of Minnesota Twin Cities, 2004 Folwell Ave, Saint Paul, Minnesota, USA.

^2^Division of Molecular Pathology and Genomics, Department of Laboratory Medicine and Pathology, University of Minnesota Twin Cities, 420 Delaware Street SE, Minneapolis, Minnesota, USA.

^3^Department of Microbiology and Immunology, University of Minnesota Medical School, 689 23rd Ave SE, Minneapolis, Minnesota, USA.

^4^Department of Veterinary Population Medicine, University of Minnesota Twin Cities, 1333 Gortner Ave., Saint Paul, Minnesota, USA.

**Supplementary Information**


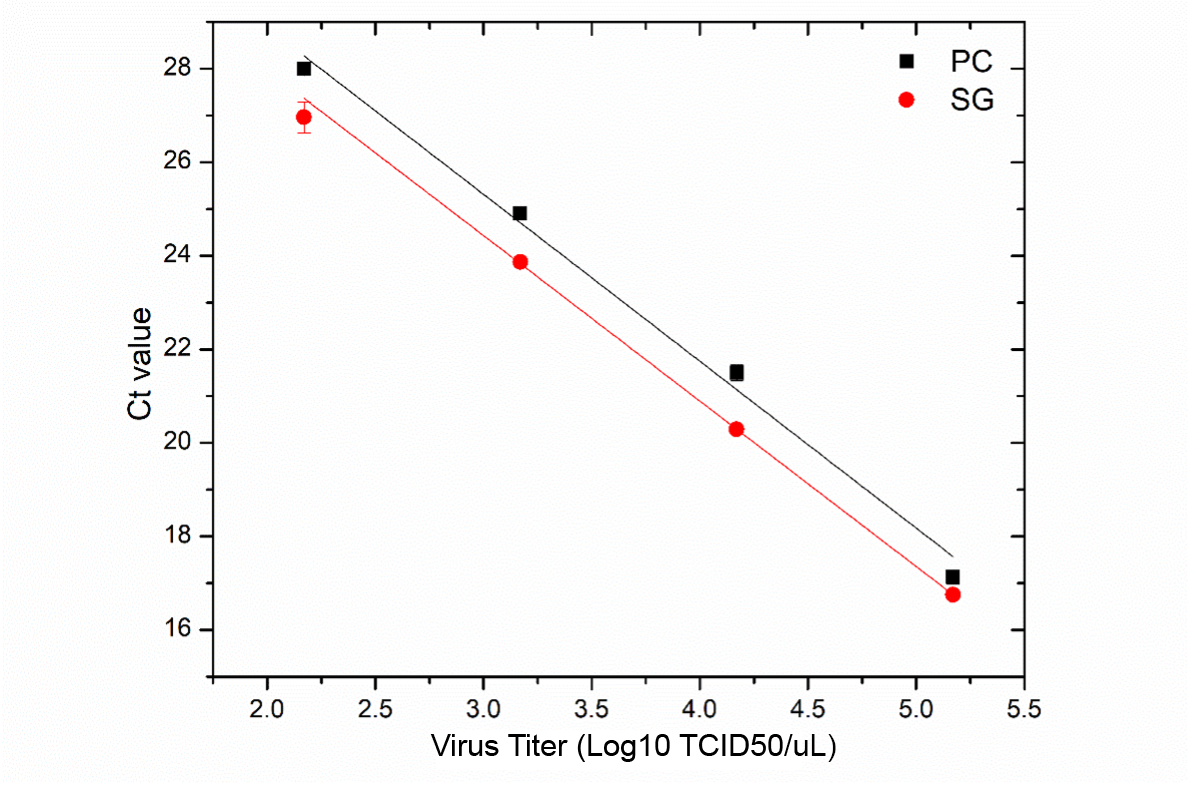


**Figure a.** A laboratory comparative study comparing the Ct value obtained from PC and SG kits. The experiment was performed on the animal coronavirus, TGEV.

**Table a.** *RT-PCR results of the preliminary experiment with 16 samples (14 positives and 2 negatives)* *using SG and PC RNA extraction kits.*

|  | **PC-based kit** | | | | **SG-based kit** | | | | |
| --- | --- | --- | --- | --- | --- | --- | --- | --- | --- |
| Sample # | N1 (PC kit) | N2 (PC kit) | RP (PC kit) | Match expected | Sample # | N1 (SG kit) | N2 (SG kit) | RP (SG kit) | Match expected |
| 1 | 31.69 | 31.84 | 35.65 | **Yes** | 1 | 29.95 | 30.82 | 29.76 | **Yes** |
| 2 | 45.00 | 37.94 | 34.80 | Yes * | 2 | 45.00 | 45.00 | 30.43 | No |
| 3 | 45.00 | 45.00 | 28.38 | No | 3 | 45.00 | 45.00 | 26.35 | No |
| 4 | 38.77 | 45.00 | 33.77 | Yes * | 4 | 45.00 | 39.08 | 31.03 | Yes * |
| 5 | 33.47 | 34.01 | 30.75 | **Yes** | 5 | 32.19 | 33.46 | 28.24 | **Yes** |
| 6 | 31.97 | 33.34 | 35.08 | **Yes** | 6 | 29.04 | 29.71 | 27.99 | **Yes** |
| 7 | 37.07 | 38.20 | 28.59 | **Yes** | 7 | 36.11 | 37.74 | 25.44 | **Yes** |
| 8 | 22.51 | 22.68 | 29.71 | **Yes** | 8 | 23.13 | 23.62 | 28.65 | **Yes** |
| 9 | 38.38 | 45.00 | 30.46 | Yes * | 9 | 45.00 | 45.00 | 28.26 | Yes * |
| 10 | 33.46 | 34.41 | 28.43 | **Yes** | 10 | 33.43 | 35.11 | 26.57 | **Yes** |
| 11 | 33.06 | 33.36 | 32.17 | **Yes** | 11 | 32.83 | 33.51 | 31.35 | **Yes** |
| 12 | 25.75 | 26.14 | 25.02 | **Yes** | 12 | 27.79 | 28.19 | 26.70 | **Yes** |
| 13 | 45.00 | 39.35 | 34.13 | Yes * | 13 | 45.00 | 45.00 | 31.92 | Yes * |
| 14 | 29.25 | 29.32 | 31.10 | **Yes** | 14 | 29.38 | 30.03 | 29.76 | **Yes** |
| 15 | Undetected | Undetected | 30.83 | **Yes** | 15 | Undetected | Undetected | 30.13 | **Yes** |
| 16 | Undetected | Undetected | 31.50 | **Yes** | 16 | Undetected | Undetected | 29.35 | **Yes** |

**Table b.** *RT-PCR results of the full-scale experiment with 64 samples (32 positives and 32 negatives)* *using SG and PC RNA extraction kits.*

|  | **Well** | **PC kit N1 Ct** | **SG kit N1 Ct** | **PC kit N2 Ct** | **SG kit N2 Ct** | **PC kit RP Ct** | **SG kit RP Ct** |
| --- | --- | --- | --- | --- | --- | --- | --- |
| Positive 1 | A01 | 45 | 45 | 38.701 | 37.802 | 32.845 | 29.166 |
| Positive 2 | A02 | 27.07 | 26.459 | 28.357 | 27.372 | 32.671 | 33.549 |
| Positive 3 | A03 | 37.622 | 30.656 | 31.894 | 29.477 | 29.118 | 29.067 |
| Positive 4 | A04 | 25.985 | 21.185 | 26.544 | 21.944 | 32.052 | 27.673 |
| Positive 5 | A05 | 45 | 45 | 45 | 45 | 30.621 | 29.976 |
| Positive 6 | A06 | 24.03 | 23.492 | 24.184 | 22.357 | 35.157 | 34.119 |
| Positive 7 | A07 | 20.949 | 19.885 | 20.725 | 20.611 | 30.241 | 28.007 |
| Positive 8 | A08 | 16.633 | 16.619 | 17.236 | 17.234 | 22.966 | 23.07 |
| Positive 9 | A09 | 21.122 | 21.075 | 21.353 | 21.378 | 26.961 | 28.942 |
| Positive 10 | A10 | 17.199 | 16.137 | 17.156 | 16.406 | 26.676 | 24.24 |
| Positive 11 | A11 | 22.197 | 22.116 | 22.472 | 22.705 | 29.869 | 27.739 |
| Positive 12 | A12 | 27.72 | 26.752 | 27.793 | 27.214 | 33.821 | 31.158 |
| Positive 13 | B01 | 28.991 | 30.029 | 29.499 | 30.613 | 31.474 | 29.551 |
| Positive 14 | B02 | 40.206 | 45 | 36.268 | 37.134 | 34.154 | 45 |
| Positive 15 | B03 | 18.17 | 17.238 | 18.61 | 17.689 | 28.68 | 24.988 |
| Positive 16 | B04 | 45 | 45 | 36.757 | 37.431 | 32.271 | 31.376 |
| Positive 17 | B05 | 21.419 | 20.139 | 21.4 | 20.794 | 27.271 | 25.366 |
| Positive 18 | B06 | 22.833 | 22.321 | 22.889 | 23.047 | 32.13 | 29.828 |
| Positive 19 | B07 | 28.985 | 27.949 | 29.223 | 28.321 | 37.19 | 33.469 |
| Positive 20 | B08 | 41.035 | 45 | 36.213 | 36.118 | 25.735 | 25.515 |
| Positive 21 | B09 | 17.027 | 16.907 | 16.776 | 17.112 | 28.99 | 27.733 |
| Positive 22 | B10 | 23.929 | 22.586 | 23.933 | 23.139 | 34.512 | 29.663 |
| Positive 23 | B11 | 45 | 45 | 36.493 | 37.771 | 29.674 | 27.246 |
| Positive 24 | B12 | 28.836 | 27.508 | 29.239 | 28.18 | 32.157 | 29.401 |
| Positive 25 | C01 | 31.374 | 31.816 | 31.843 | 31.569 | 31.774 | 29.911 |
| Positive 26 | C02 | 20.97 | 19.878 | 21.736 | 20.195 | 31.893 | 29.198 |
| Positive 27 | C03 | 24.704 | 24.049 | 25.198 | 24.585 | 32.018 | 29.096 |
| Positive 28 | C04 | 19.685 | 17.637 | 19.279 | 18.177 | 32.565 | 27.834 |
| Positive 29 | C05 | 45 | 45 | 45 | 45 | 34.637 | 31.49 |
| Positive 30 | C06 | 38.176 | 32.068 | 31.266 | 30.619 | 31.764 | 29.663 |
| Positive 31 | C07 | 31.688 | 31.205 | 31.212 | 31.399 | 33.296 | 29.813 |
| Positive 32 | C08 | 31.443 | 29.734 | 31.479 | 30.435 | 29.483 | 26.678 |
|  |  |  |  |  |  |  |  |
| Negative 1 | C09 | 45 | 45 | 45 | 45 | 32.749 | 31.069 |
| Negative 2 | C10 | 45 | 45 | 45 | 45 | 33.486 | 30.574 |
| Negative 3 | C11 | 45 | 45 | 45 | 45 | 34.063 | 32.185 |
| Negative 4 | C12 | 45 | 45 | 45 | 45 | 34.738 | 33.849 |
| Negative 5 | D01 | 45 | 45 | 45 | 37.756 | 33.201 | 31.193 |
| Negative 6 | D02 | 45 | 45 | 45 | 37.717 | 33.907 | 32.749 |
| Negative 7 | D03 | 45 | 45 | 45 | 45 | 35.584 | 34.098 |
| Negative 8 | D04 | 45 | 45 | 45 | 38.753 | 34.996 | 32.45 |
| Negative 9 | D05 | 45 | 45 | 45 | 45 | 29.864 | 26.658 |
| Negative 10 | D06 | 45 | 45 | 45 | 45 | 32.712 | 30.552 |
| Negative 11 | D07 | 45 | 45 | 45 | 45 | 33.985 | 31.924 |
| Negative 12 | D08 | 45 | 45 | 45 | 45 | 30.399 | 29.21 |
| Negative 13 | D09 | 45 | 45 | 45 | 45 | 32.979 | 31.37 |
| Negative 14 | D10 | 45 | 45 | 45 | 45 | 34.62 | 31.669 |
| Negative 15 | D11 | 45 | 45 | 45 | 45 | 33.55 | 32.394 |
| Negative 16 | D12 | 45 | 45 | 45 | 45 | 29.859 | 29.374 |
| Negative 17 | E01 | 45 | 45 | 45 | 45 | 30.424 | 27.436 |
| Negative 18 | E02 | 45 | 45 | 45 | 45 | 30.101 | 28.34 |
| Negative 19 | E03 | 45 | 45 | 45 | 45 | 30.276 | 28.151 |
| Negative 20 | E04 | 45 | 45 | 45 | 45 | 34.71 | 32.363 |
| Negative 21 | E05 | 45 | 45 | 45 | 45 | 30.687 | 29.377 |
| Negative 22 | E06 | 45 | 45 | 45 | 45 | 33.196 | 30.332 |
| Negative 23 | E07 | 45 | 45 | 45 | 45 | 34.261 | 31.769 |
| Negative 24 | E08 | 45 | 45 | 45 | 45 | 34.192 | 31.513 |
| Negative 25 | E09 | 45 | 45 | 45 | 45 | 34.27 | 31.026 |
| Negative 26 | E10 | 45 | 45 | 45 | 45 | 35.019 | 31.885 |
| Negative 27 | E11 | 45 | 45 | 45 | 45 | 32.318 | 30.017 |
| Negative 28 | E12 | 45 | 45 | 45 | 45 | 35.215 | 32.169 |
| Negative 29 | F01 | 45 | 45 | 45 | 45 | 29.621 | 28.083 |
| Negative 30 | F02 | 45 | 45 | 45 | 38.372 | 33.929 | 31.225 |
| Negative 31 | F03 | 45 | 45 | 45 | 45 | 33.304 | 30.517 |
| Negative 32 | F04 | 45 | 45 | 45 | 38.997 | 35.317 | 33.32 |

**Table c.** *Raw data of the experiment evaluating the limit of detection for the SG and PC kits.*

| **PC kit** | | | | | **SG kit** | | | | |
| --- | --- | --- | --- | --- | --- | --- | --- | --- | --- |
| **Well** | **Cp/µl** | **N1 Ct** | **N2 Ct** | **RP Ct** | **Well** | **Cp/µl** | **N1 Ct** | **N2 Ct** | **RP Ct** |
| A01 | 0 | Undetected | Undetected | Undetected | B07 | 0 | Undetected | Undetected | Undetected |
| A02 | 90 | 30.92 | 31.16 | 30.28 | B08 | 90 | 30.37 | 30.79 | 29.59 |
| A03 | 90 | 30.84 | 31.32 | 30.43 | B09 | 90 | 30.55 | 30.97 | 29.84 |
| A04 | 90 | 31.09 | 31.56 | 31.27 | B10 | 90 | 30.59 | 31.04 | 29.72 |
| A05 | 45 | 32.03 | 32.49 | 32.4 | B11 | 45 | 31.31 | 32.07 | 30.6 |
| A06 | 45 | 32.14 | 32.76 | 32.64 | B12 | 45 | 31.17 | 31.48 | 30.01 |
| A07 | 45 | 32.18 | 32.72 | 31.97 | C01 | 45 | 31.22 | 31.92 | 30.33 |
| A08 | 15 | 33.59 | 34.42 | 33.26 | C02 | 15 | 32.58 | 33.13 | 31.96 |
| A09 | 15 | 34.2 | 34.77 | 33.65 | C03 | 15 | 33.25 | 33.76 | 31.98 |
| A10 | 15 | 33.94 | 35.01 | 33.43 | C04 | 15 | 33.06 | 33.99 | 31.89 |
| A11 | 5 | 35.47 | 35.33 | 35.04 | C05 | 5 | 34.57 | 35.27 | 33.04 |
| A12 | 5 | 34.95 | 36.19 | 35.36 | C06 | 5 | 34.45 | 35.28 | 33.24 |
| B01 | 5 | 35.42 | 36.68 | 35.27 | C07 | 5 | 36.38 | 35.37 | 33.75 |
| B02 | 1.67 | 37.93 | 37.61 | 37.96 | C08 | 1.67 | 36.07 | Undetected | 35.08 |
| B03 | 0.56 | Undetected | Undetected | Undetected | C09 | 0.56 | 37.18 | Undetected | 36.04 |
| B04 | 0.19 | Undetected | Undetected | Undetected | C10 | 0.19 | Undetected | Undetected | 38.25 |
| B05 | 0 | Undetected | Undetected | Undetected | C11 | 0 | Undetected | Undetected | Undetected |
| B06 | 0 | Undetected | Undetected | 34.77 | C12 | 0 | Undetected | Undetected | 32.5 |

| Table d. *Oligonucleotides for TaqMan-based RT-qPCR used in this study.* a: +, virus sense; −, anti-virus sense, b: Corresponding nucleotide position of TGEV (GenBank accession no.: KX900410.1) as reference. | | | | | | |
| --- | --- | --- | --- | --- | --- | --- |
| Name of Primer or Probe | **Oligonucleotide name** | **Sequence (5’ - 3’)** | **Polarity^a^** | **Position** | **Product length (pb)** | **Reference** |
| TGEV RT-qPCR | TGEV-F | TCTGCTGAAGGTGCTATTATATGC | + | 20722 – 20745^b^ | 146 | 14 |
|  | TGEV-R | CCACAATTTGCCTCTGAATTAGAAG | - | 20867 - 20843^b^ |  |  |
|  | TGEV-P | FAM-TAAGGGCTC/ZEN/ACCACCTACTACCACCA-3IABkFQ | + | 20751- 20776^b^ |  |  |
| TGEV RT-PCR  (For standard curve construction) | TGEV-F2 | GCAGGTTACCACCTAATTCAGA | + | 20486-20507^b^ | 557 | In-house designed |
|  | TGEV-R2 | CAGGATTAAACCACCAAAGGTC | - | 21043-21022^b^ |  |  |

**
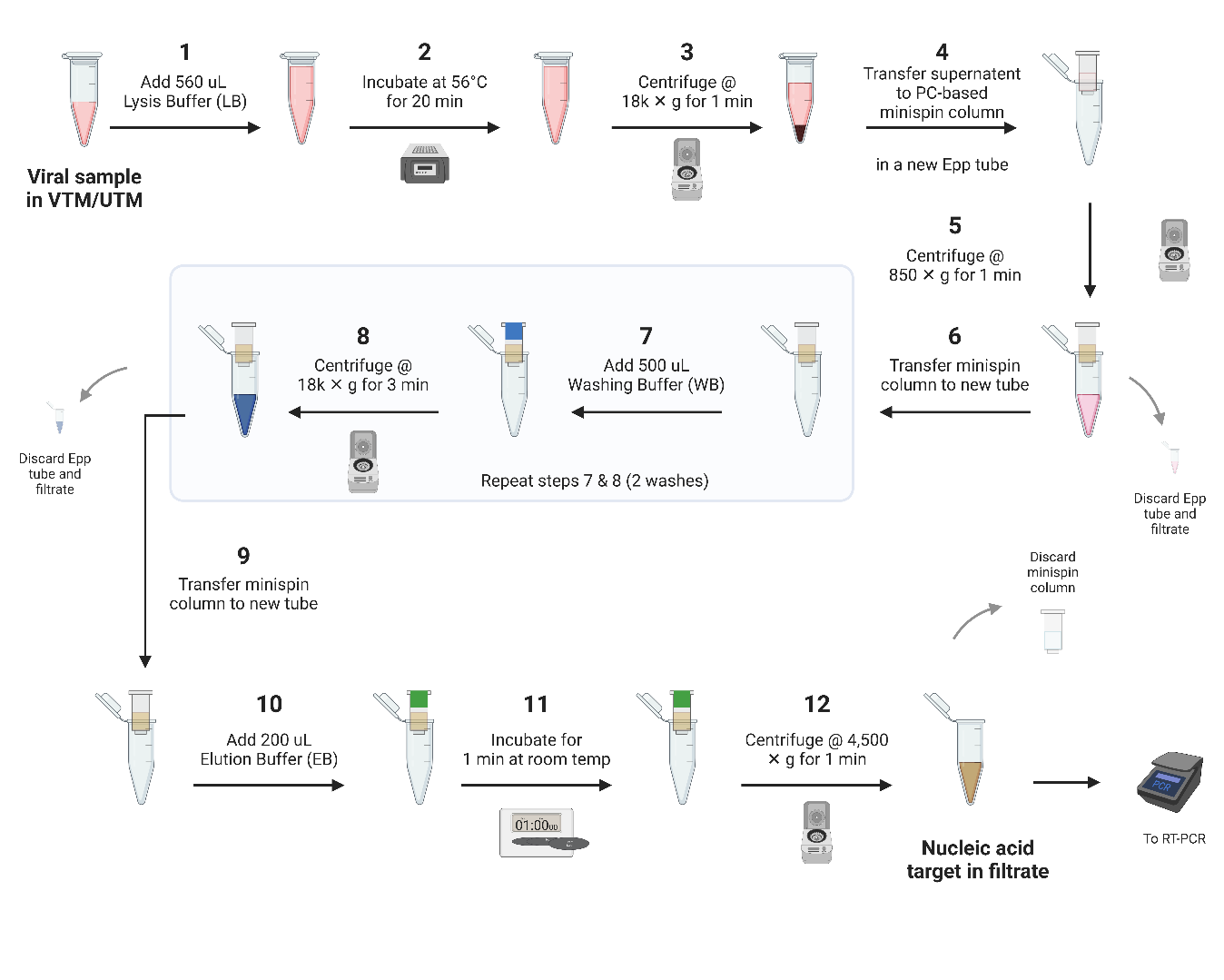
**

**Figure b.** Graphical prep protocol for RAPID. Figure created using BioRender.com.
